# Construction of a prognosis-predicting model based on autophagy-related genes for hepatocellular carcinoma (HCC) patients

**DOI:** 10.18632/aging.103507

**Published:** 2020-07-18

**Authors:** Yayun Zhu, Ru Wang, Wanbin Chen, Qiuyu Chen, Jian Zhou

**Affiliations:** 1 Department of Liver Surgery and Transplantation, Liver Cancer Institute, Zhongshan Hospital, Fudan University, Shanghai, China; 2 Institute for Cell Engineering, The Johns Hopkins University School of Medicine, Baltimore, MD 21205, USA; 3 Department of Breast Surgery, The First Affiliated Hospital of Xi’an Jiaotong University, Xi’an, Shaanxi, China; 4 Department of Marketing, The Johns Hopkins University Carey Business School, Baltimore, MD 21202, USA; 5 Department of Clinical Immunology, Children’s Hospital of Fudan University, Shanghai, China; 6 Department of Liver Surgery and Transplantation, Liver Cancer Institute and State Key Laboratory of Genetic Engineering, Fudan University, Shanghai, China; 7 Key Laboratory of Carcinogenesis and Cancer Invasion, Fudan University, Ministry of Education, Shanghai, China; 8 Institute of Biomedical Sciences, Fudan University, Shanghai, China; 9 Shanghai Key Laboratory of Organ Transplantation, Zhongshan Hospital, Fudan University, Shanghai, China

**Keywords:** autophagy-related genes, prognosis, hepatocellular carcinoma, The Cancer Genome Atlas

## Abstract

Background: Autophagy, a highly conserved cellular catabolic process by which the eukaryotic cells deliver autophagosomes engulfing cellular proteins and organelles to lysosomes for degradation, is critical for maintaining cellular homeostasis in response to various signals and nutrient stresses. The dysregulation of autophagy has been noted in the pathogenesis of cancers. Our study aims to investigate the prognosis-predicting value of autophagy-related genes (ARG) in hepatocellular carcinoma (HCC).

Results: The signature was constructed based on eight ARGs, which stratified HCC patients into high- and low-risk groups in terms of overall survival (OS) (Hazard Ratio, HR=4.641, 95% Confidential Interval, CI, 3.365-5.917, P=0.000). The ARG signature is an independent prognostic indicator for HCC patients (HR = 1.286, 95% CI, 1.194-1.385; P < 0.001). The area under the receiver operating characteristic (ROC) curve (AUC) for 5-year survival is 0.765.

Conclusion: This study provides a potential prognostic signature for predicting the prognosis of HCC patients and molecular insights into the significance of autophagy in HCC.

Methods: Sixty-two differentially expressed ARGs and the clinical characteristics and basic information of the 369 enrolled HCC patients were retrieved from The Cancer Genome Atlas (TCGA) database. the Cox proportional hazard regression analysis was adopted to identify survival-related ARGs, based on which a prognosis predicting signature was constructed.

## INTRODUCTION

Hepatocellular carcinoma (HCC), the fourth leading cause of all cancer-related deaths worldwide, accounts for 90% of all primary liver malignancies [1], the major attributable factors of which are chronic hepatitis B or C virus infection and the abuse of alcohol [2].

Current antiviral drugs and surgical interventions, along with immunotherapeutic agents and targeted therapy, are improving HCC patients’ survival outcomes in an inspiring way, which, however, practically are often challenged by the tumor heterogeneity [3] and the development of drug resistance [4, 5].

Also, the lack of a robust model predicting the prognosis and the occurrence of resistance during courses of therapies contributes to poor survival outcomes.

Recent advancements in the next-generation sequencing technology have characterized the genetic landscape of various types of cancer, including HCC, not only revealing the driver mutations in hotspot genes like TP53, CTNNB1, TERT promoter but identifying the dysregulated expression of genes related to diverse pathways in multiple biological processes such as the metabolic pathways, VHL/HIF oxygen-sensing pathway, the DDR pathway, and the autophagy.

Autophagy, executed by autophagy-related genes (ARG), having roles in various cellular functions in cancer, both protecting against and contributing to the proliferation of cancer cells, is a highly conserved cellular catabolic process by which the eukaryotic cells deliver autophagosomes engulfing cellular proteins and organelles to lysosomes for degradation, which is critical for maintaining cellular homeostasis in response to various signals and cellular stresses. The dysregulation of autophagy has been noted in the pathogenesis of diverse diseases, including cancers.

The conception of harnessing this pathway to improve clinical outcomes of cancer patients has been the attention of researchers seeking to redirect the upregulation of autophagy flux enabling tumor cell survival and growth since the terminological introduction of autophagy in 1963 by Christian de Duve [6].

Studies surged in the past a few decades laying groundwork for the idea that enhancing autophagy might help prevent progression of early-stage cancers [7], and that both enhancers and retarders of autophagy can bring therapeutic benefits to advanced cancers [8–11].

Increasing pre-clinical evidence from animal models and *in vitro* studies using genetically engineered mouse models and patient-derived xenografts mouse models has suggested an anti-tumor effect of inhibiting autophagy, either pharmacologically or genetically [8, 9, 12, 13].

ARGs, originally identified as the mediator of the formation of double-membrane structures delivering contents from the intra-cytoplasm to the lysosome for self-degradation, have diverse physiologically important roles in other membrane trafficking and signaling pathways [14].

The autophagy and ARGs have been reported in HCC not only as maintaining liver homeostasis, contributing to the preservation of genome stability in the liver cell, but saving normal liver cells from being transformed into cancer cells by helping clearing detrimental mitochondria and the transformed cells [15].

Adding to the evidence that autophagy plays a tumor-suppressing role in HCC was the fact that knockout of the key autophagy gene Beclin1, the only dual function molecule acting as both tumor suppressor and autophagy modulator [16], in mice model [17], led to reduced autophagy activity and increased HCC initiating ability [17], which was evidenced by more studies showing that Beclin1 was associated with HCC progression and thus could be a potential prognostic biomarker for HCC patients [18].

We explored in our study the correlation of the ARGs with clinical outcomes of 377 HCC patients from the TCGA database with a prognosis-predicting model constructed as an independent indicator of overall survival based on a signature consisting of 8 ARGs selected from the multivariate Cox regression analysis, allowing the improvement in the prognosis-predicting efficiency and accuracy for HCC patients.

## RESULTS

### Identification of differentially expressed ARGs

**Figure 1:**
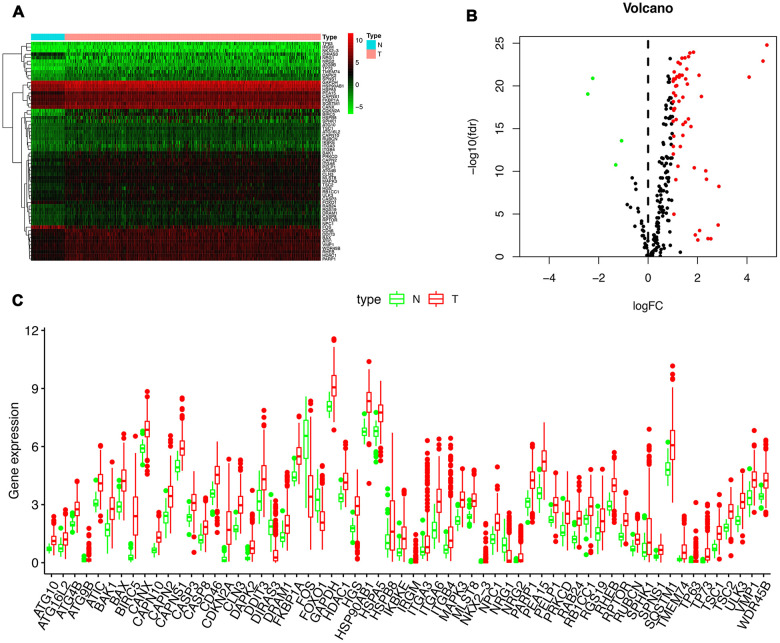
**Differentially expressed autophagy-related genes (ARGs) between liver cancer (HCC) and normal liver tissues.** Heatmap (**A**) and volcano map (**B**) were constructed showing the 62 differentially expressed autophagy-related genes in HCC tissues compared with normal tissue, with red dots representing significantly up-regulated genes, green dots representing significantly down-regulated genes, and black dots representing genes with no significant differences. (**C**) Expression of 62 ARGs that are differentially expressed in HCC tissues (each red dot represents a distinct tumor sample) as compared with the normal tissues (green dots). The upregulation of a distinct gene was marked as red bars, and the downregulation as green bars.

RNA-seq and clinical data from 23126 HCC tissue samples and 3038 non-tumor samples were downloaded from TCGA. 369 patients in total with primary HCC who were followed for more than 1 month were included in the study. The expression values of 232 ARGs were extracted. Considering the criteria for FDR <0.05 and [log2 (fold change)]> 1, we finally obtained 62 up-regulated ARGs (Figure 1A, 1B). A box plot was generated showing the expression pattern of 62 differentially expressed ARGs between HCC and non-tumor tissue (Figure 1C). Scatter plot showing expression patterns of 62 up-regulated genes.

### Functional enrichment of the differentially expressed ARGs

**Figure 2:**
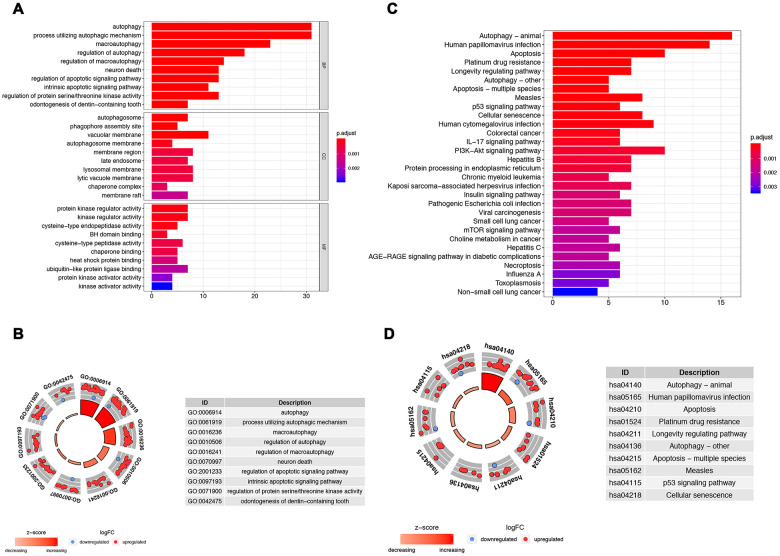
**Gene functional enrichment analysis for the ARGs.** (**A**, **B**) Show, by the GO analysis, the biological process and molecular functions that the ARGs are involved in; (**C**, **D**) Show the KEGG analysis for potential pathways by which these ARGs exert their effects on tumor cells.

Functional enrichment analysis of 62 differentially expressed ARGs provides a biological understanding of these genes. Top 30 of GO enrichment and top 30 of pathway enrichment are summarized in Figure 2. GO and KEGG analyses revealed that the ARGs were mainly involved in autophagy, apoptotic signaling pathway, regulation of protein serine/threonine kinase activity, PI3K-Akt signaling pathway, and p53 signaling pathway (Figure 2A–2D).

### Identification of prognostic ARGs

**Figure 3:**
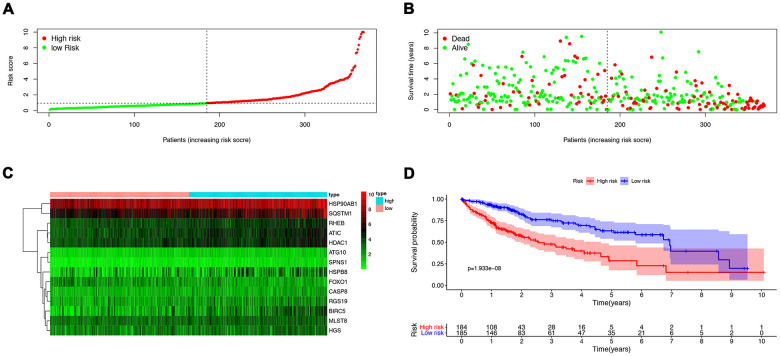
**The construction of a prognostic ARG signature.** (**A**) Distribution of prognostic index. (**B**) Survival status of patients in different groups. (**C**) Heat map of the expression profile of the included ARGs. (**D**) Patients in the high-risk group have a shorter overall survival.

**Figure 4:**
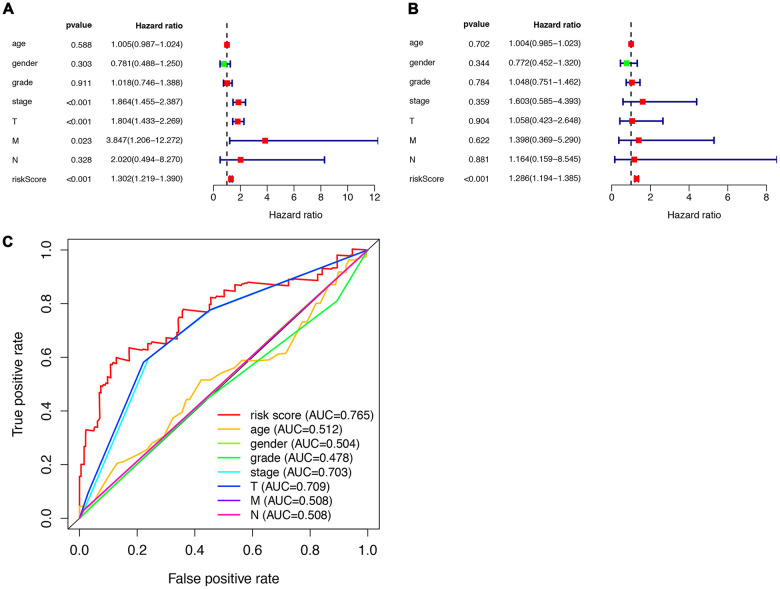
**Prognostic indicators based on ARGs show good predictive performance.** A forest plot of univariate (**A**) and multivariate (**B**) Cox regression analysis in HCC. (**C**) Survival-dependent receiver operating characteristic (ROC) curves validate the prognostic significance of ARGs-based prognostic indicators.

To analyze ARGs’ involvement in HCC progression, we screened for ARGs that were significantly associated with prognosis. The univariate Cox regression analysis indicates that 32 ARGs that are correlated with the overall survival are all risk factors (Table 1). A total of 8 genes (RHEB, HSP90AB1, ATIC, HDAC1, MLST8, SQSTM1, SPNS1, and HSPB8) were observed to be significantly associated with the OS by multivariate cox regression analysis (Table 2), based on which we constructed autophagy prognostic signature to better predict the clinical outcomes (OS) for HCC patients. Figure 3 showed the distribution of the ARG signature in the TCGA dataset (Figure 3A), survival status of patients in different groups (Figure 3B) and heatmap of the expression profile of the included ARGs (Figure 3C). To determine the performance of the signature in predicting OS in HCC patients, K-M survival curves were plotted to analyze different survival times between high-risk and low-risk groups, showing that the survival rate of patients in the high-risk group was significantly lower than that in the low-risk group (Figure 3D). Also, after adjusting for clinicopathological features such as age, tumor differentiation grade, tumor stage, tumor size, lymph node metastasis, and distal metastasis, the signature remained an independent prognostic indicator for HCC patients in univariate analysis (HR=1.302, 95% CI, 1.219-1.390; P<0.001; Figure 4A) and multivariate analysis (HR = 1.286, 95% CI, 1.194-1.385; *P* < 0.001; Figure 4B). The AUC of the ROC curve for 5-year survival is 0.765, which is much higher than that of ROC of age (0.512), gender (0.504), tumor differentiation grade (0.478), the Tumor, Nodes, and Metastases (TNM) stage (0.703), tumor size (0.709), metastatic status (0.508), and lymph node status (0.508).

**Table 1:** Univariate cox regression analysis identified 32 ARGs related to the HCC risks.

**Genes**	**HR**	**95% CI**	**p value**
IKBKE	1.35	1.10-1.66	0.004
RHEB	1.91	1.36-2.70	0.000
CAPN10	2.59	1.66-4.04	0.000
GAPDH	1.49	1.20-1.86	0.000
HSP90AB1	1.39	1.09-1.78	0.007
ATG10	2.05	1.24-3.39	0.005
CDKN2A	1.24	1.07-1.44	0.004
NPC1	1.78	1.36-2.34	0.000
PEA15	1.37	1.10-1.72	0.006
FKBP1A	1.65	1.27-2.14	0.000
ATIC	1.9	1.45-2.48	0.000
HDAC1	1.93	1.45-2.56	0.000
RAB24	1.73	1.24-2.42	0.001
BIRC5	1.34	1.17-1.54	0.000
MLST8	1.41	1.02-1.95	0.036
SQSTM1	1.38	1.17-1.62	0.000
CASP8	1.61	1.15-2.27	0.006
MAPK3	1.56	1.17-2.09	0.003
CANX	1.36	1.05-1.78	0.022
RGS19	1.39	1.10-1.77	0.007
FOXO1	0.74	0.58-0.94	0.016
BAK1	1.38	1.13-1.69	0.002
ATG4B	1.56	1.09-2.22	0.015
TSC1	1.45	1.00-2.08	0.048
SPNS1	2.61	1.54-4.43	0.000
HSPB8	1.15	1.04-1.28	0.008
TMEM74	1.54	1.11-2.15	0.011
WDR45B	1.51	1.14-2.00	0.004
RUBCN	2.31	1.48-3.61	0.000
HGS	1.33	1.05-1.68	0.018
PRKCD	1.55	1.25-1.91	0.000
DRAM1	1.28	1.04-1.59	0.022

**Abbreviations:** HR, Hazardous Ratio; CI, Credential Interval.

**Table 2:** Multivariate cox regression analysis identified 8 ARGs that are independent factors for HCC risks.

**Genes**	**Co-efficient**	**HR**	**95% CI**	**p value**
RHEB	0.53	1.70	1.15-2.51	0.01
HSP90AB1	-0.29	0.75	0.56-0.99	0.05
ATG10	0.40	1.49	0.87-2.55	0.15
ATIC	0.66	1.94	1.31-2.88	0.00
HDAC1	0.47	1.59	1.10-2.30	0.01
BIRC5	0.18	1.20	0.99-1.47	0.07
MLST8	-0.76	0.47	0.30-0.74	0.00
SQSTM1	0.23	1.26	1.03-1.54	0.03
CASP8	-0.47	0.63	0.38-1.03	0.07
RGS19	-0.26	0.77	0.56-1.07	0.12
FOXO1	-0.26	0.77	0.57-1.06	0.11
SPNS1	1.62	5.05	2.09-12.17	0.00
HSPB8	0.15	1.17	1.02-1.33	0.02
HGS	-0.36	0.70	0.48-1.03	0.07

**Abbreviations:** HR, Hazardous Ratio; CI, Credential Interval.

This indicated that the prognostic index based on ARGs has a certain potential in survival prediction (Figure 4C).

### Prognostic significance of the ARG signature

**Figure 5:**
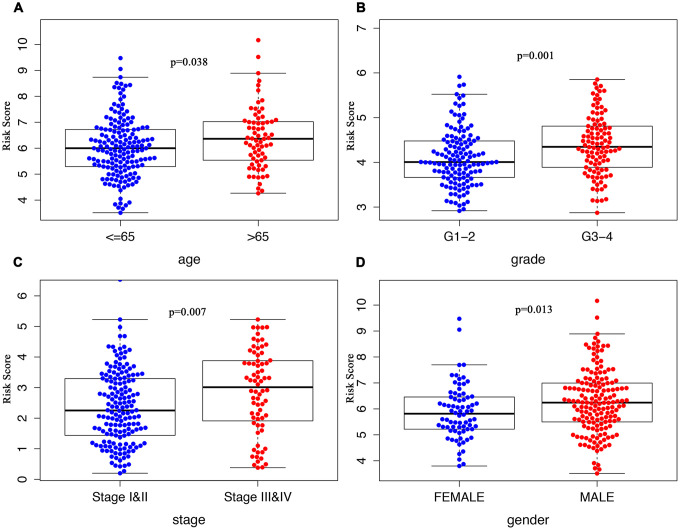
**Clinicopathological significance of the ARG signature in HCC.** Risk scores among different clinical features in HCC. P values were all less than 0.05 for (**A**) age, grade (**B**), TNM stage (**C**), and gender (**D**) between groups.

The clinical significance of the signature was assessed by analyzing its correlation with the clinical parameters, which suggested significantly increased risk score in patients who were older than 65 (Figure 5A), in Grade III/IV tumor differentiation (Figure 5B), in TNM stage III/IV (Figure 5C), or males (Figure 5D).

## DISCUSSION

Despite the numerousness of studies reporting that autophagy is not only involved in the initiation but the progression and drug resistance of HCC, the specific functions of ARGs and their clinical significance in HCC has not been exploited and clarified well yet [14, 19–22]. The exploration of autophagy and ARGs has been tapping into the developing effective biomarkers for HCC prognosis-predicting and therapy monitoring, single ARG at a time. The limitation of a single autophagy gene in predicting the survival outcomes warrants an expansion of the ARG list that are potentially applicable for predicting the prognosis of HCC patients. We finally identified 8 prognostic ARGs from screening from a total number of 237 ARGs from the ATCG datasets for establishing a prognostic model that might be used for prognosis prediction in HCC patients, which offers a new perspective into the scheming of developing individualized therapy options based on a prognostic stratification via this model.

The model we constructed consists of a group ARGs that are correlated with the survival outcomes of HCC patients confirmed by the univariate and then the multivariate cox regression analysis.

Most of the genes incorporated for the construction of the signature were explored in previous studies as a progression promoter and a prognosis indicator for patients with HCC or other malignancies. The knockdown of RHEB, a key regulator in mTOR signaling pathway [23], was related with an inhibited effect on the growth of SMMC-7721 cells, and the upregulated expression of RHEB in human HCC tissues was correlated poorer prognosis as compared with those who had lower expression level of RHB [24–29]. HSP90AB1, also referred to as HSP90β, was reported to promote the angiogenesis [30–33] by activating VEGFRs transcription in an epithelial cell-dependent way or by interacting with Twist1, increasing nuclear translocation, and activating VE-cadherin transcription to induce EMT in HCC, suggesting that HSP90β might be a novel target for antitumor therapy. ATIC, likewise, which, however, has only taken into consideration a was identified as an oncogenic gene promoting cell survival, proliferation, and migration by targeting the AMPK-mTOR-S6 K1 pathway [34], the aberrantly upregulated expression of which was correlated with poor survival of HCC patients. Transient knockdown of ATIC by siRNA partially impaired DNA double-strand break repair, shortening cellular survival following radiation, which implied that targeting ATIC may be an effective chemoradiotherapy sensitizer [35]. Also, the rest of the genes in the signature, including HDAC1, MLST8, SQSTM1, CASP8, RGS19, FOXO1, SPNS1, and HSPB8, were all reported to promote tumor progression either via targeting the mTOR pathway [36], the metabolism pathway [37], the cAMP/PKA/CREB [38] or Akt pathway [39–41] in various types of malignancies, including HCC. Based on the extensive literature evidencing the notion that the ARGs are clinically relevant to the prognostic outcomes of HCC patients and could be potentially used as biomarkers for both monitoring treatments and predicting prognosis.

Limitations of this study are:1) Data in our study were collected retrospectively, leaving some internal bias inevitable; 2) The prognostic signature established in our study needs further validation from more independent studies to make the signature more convincing; 3) Experimental explorations into the molecular mechanism underlying the functions of these genes.

In summary, we first demonstrated the clinical significance of an ARG signature in predicting the overall survival of HCC patients. The ARGs were identified to be involved in HCC growth and progression through different pathways mentioned above. Adding to the reliability of the ARG signature is the consistency of our findings with previous studies showing that some of the ARGs are capable of forecasting the survival outcomes and monitoring tumor progression and treatment responses. Therefore, presumably the ARGs identified either in our study or elsewhere might hold promise as a novel biomarker for human HCC therapy, making the development and test of the effect of ARG inhibitors clinically desirable. It’s also of great interest to unravel the underlying molecular mechanism of these genes and their roles in other types of malignancies.

## CONCLUSIONS

In conclusion, our study demonstrates for the first time the potential prognostic role of an ARG signature in HCC, although more in-depth mechanisms and prognostic roles for this signature in HCC need to be confirmed in the future, our findings provide a preliminary basis to explore ARGs as a potential molecular target for the development of HCC therapies.

## MATERIALS AND METHODS

### Data retrieval

To identify the ARGs that could predict the prognosis of HCC patients, we retrieved the mRNA expression data of 377 HCC patients from the Cancer Genome Atlas (TCGA) datasets. RNA-seq data of 221 ARGs and the clinical features of the patients were obtained for further analysis.

### Identification of differentially expressed ARGs by enrichment analysis

Differentially expressed ARGs were identified from a list of total 632 ARGs in HCC from the TCGA datasets by using the EdgeR package in R statistical software. ARGs were only considered as differentially expressed ARGs when at least 2-fold change, evidenced by a p-value less than 0.05, in the expression level was observed. Gene functional enrichment analyses, using gene ontology (GO) and Kyoto Encyclopedia of Genes and Genomes (KEGG), was conducted based on the differentially expressed ARGs to find the major functional and molecular attributes of these genes.

### Construction of an individualized prognostic index based on ARGs

The prognosis-predicting model based on ARGs was constructed using the multivariate cox regression analysis. A scoring formula for each HCC patient was established and weighted by its expected regression coefficients in a multivariate cox regression analysis after including the expression values for every single gene and was then employed to determine the use of the median risk score as the cut-off point characterize patients into a high-risk group and low-risk group. The Kaplan-Meier estimator and the multivariate cox regression analysis were adopted to assess the differences in survival in these two groups, and the role of risk scores in predicting survival outcomes for HCC patients, respectively. ROC curves were used to study the accuracy of the prediction model.

### Statistical analysis

Statistical analyses were performed mostly based on the R 3.5.1(https://www.r-project.org/). The univariate Cox regression analysis was used to evaluate the association between gene expression profiles and overall survival.

Differences between survival curves generated by the Kaplan-Meier method was defined by log-rank tests.

The Multivariate Cox regression model was employed to construct the model based on the factor correlated with survival.

The package of “survival ROC” built-in R was utilized to generate the receiver operating characteristic (ROC) curve and calculate the area under the ROC curve (AUC) for each dataset to measure the prognostic role of the model.

All statistical tests were only considered significant when p <0.05 was achieved.
